# Supplementary material for: Transcriptome analysis of Citrus Aurantium L. to study synephrine biosynthesis during developmental stages
Source: PeerJ. 2024 Sep 9;12:e17965. doi: 10.7717/peerj.17965 (PMC11391941; doi:10.7717/peerj.17965)
Supplement: Supplemental Information 4 [file peerj-12-17965-s004.docx]

**
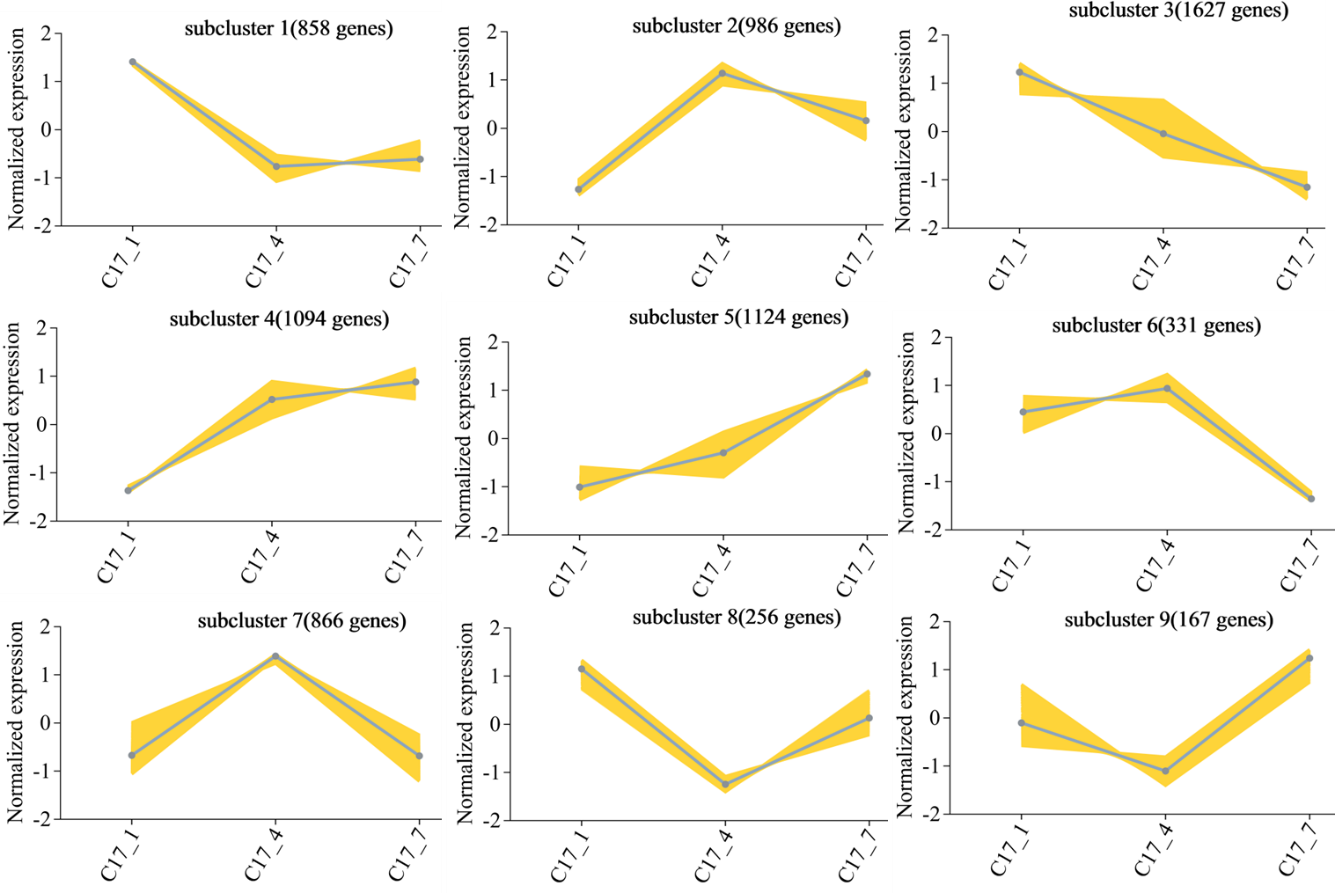
**

**Fig S1 Based on the H-cluster, K-means, and SOM methods, differentially expressed genes (DEGs) were clustered by log2 (ratios) of their relative expression level. Multiple clusters were formed from the DEGs using various clustering approaches. The clustering graph's yellow line represents the relative expression of genes inside a cluster at different developmental stages based on the expression level of the initial sample. The blue line shows the cluster's average relative expression of all genes at each developmental stage.**

**
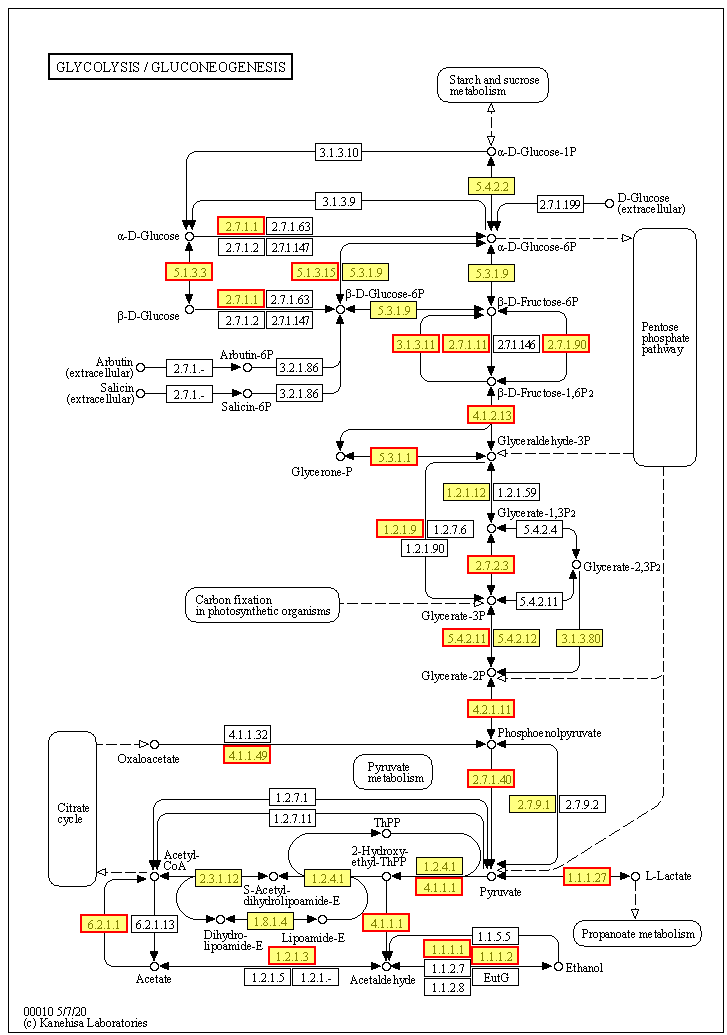
**

**Fig S2 Glycolysis/gluconeogenesis biosynthesis. Yellow background genes inside the red boxes in each metabolic pathway indicate significant up-regulation of the pairwise compared samples at the different growth stages, while green background genes show significant down-regulation.**

**
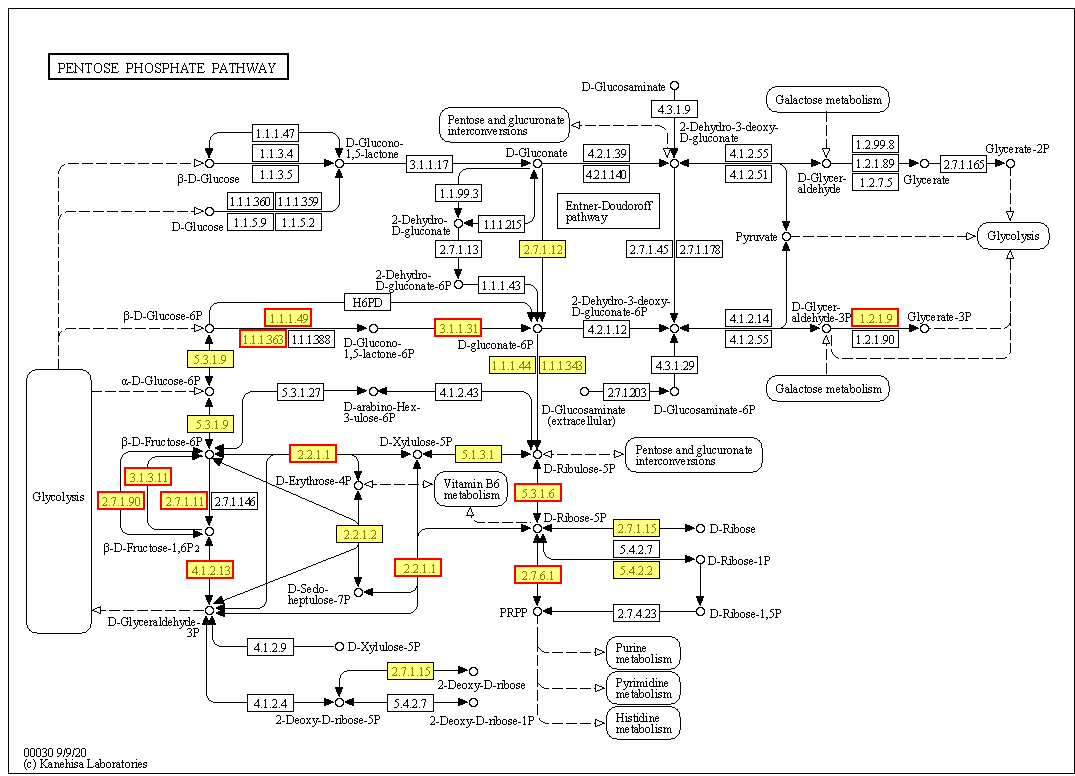
**

**Fig S3 Pentose phosphate biosynthesis. Yellow background genes inside the red boxes in each metabolic pathway indicate significant up-regulation of the pairwise compared samples at the different growth stages, while green background genes show significant down-regulation.**

**
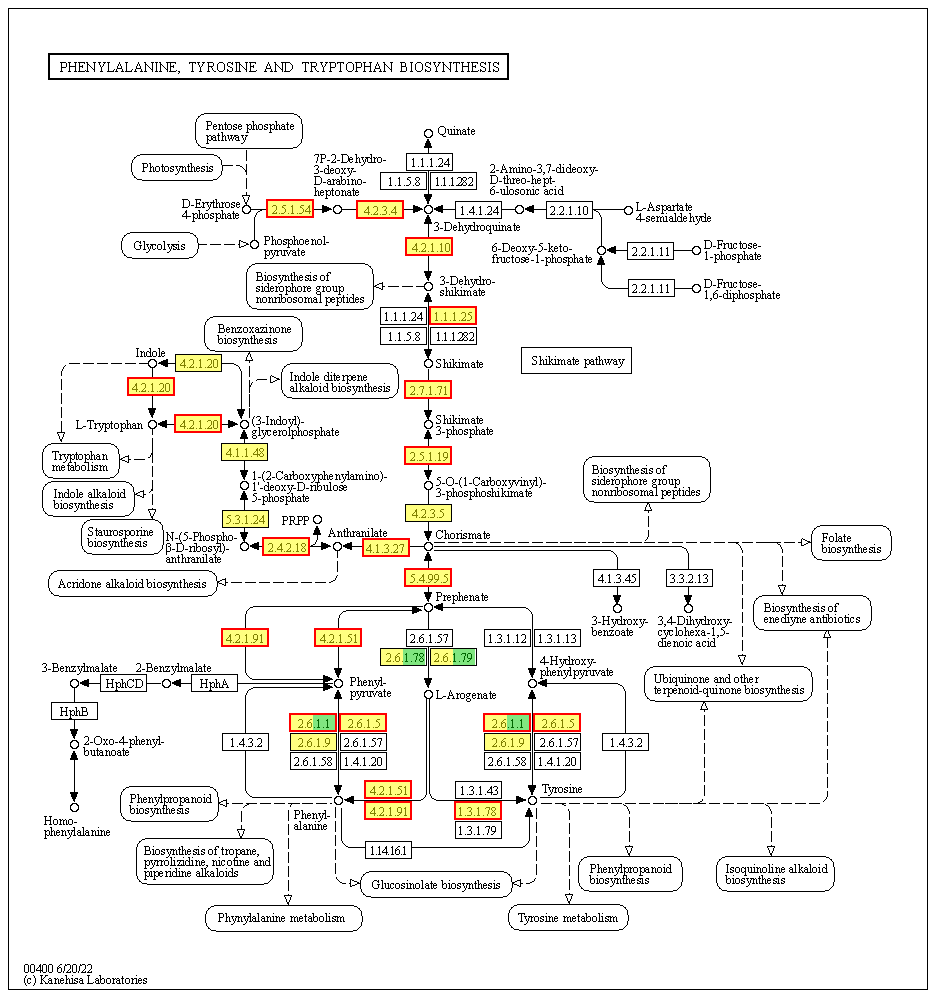
**

**Fig S4 Phenylalanine, tyrosine and tryptophan biosynthesis. Yellow background genes inside the red boxes in each metabolic pathway indicate significant up-regulation of the pairwise compared samples at the different growth stages, while green background genes show significant down-regulation.**

**
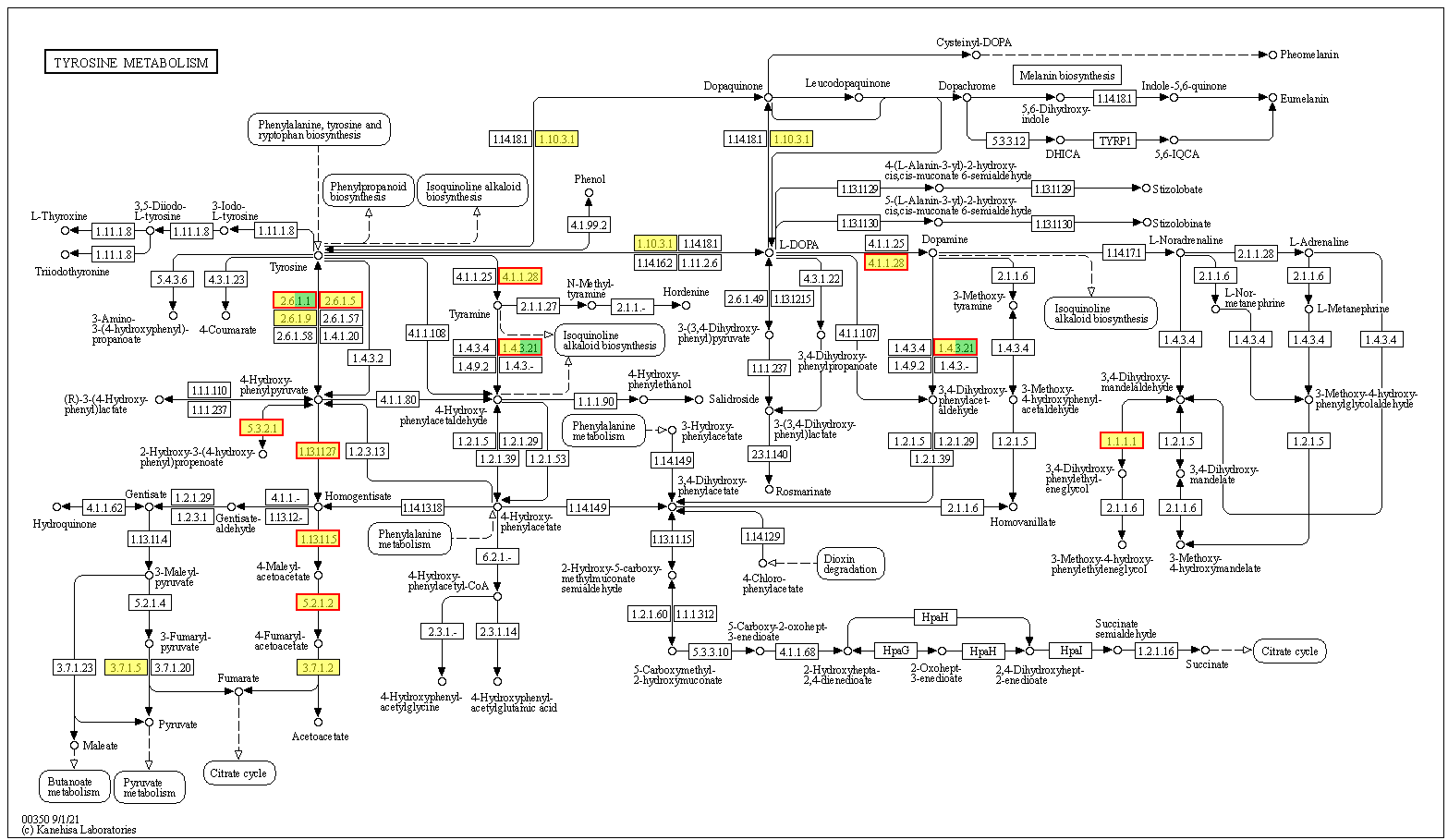
**

**Fig S5 Tyrosine metabolism.** **Yellow background genes inside the red boxes in each metabolic pathway indicate significant up-regulation of the pairwise compared samples at the different growth stages, while green background genes show significant down-regulation.**

**
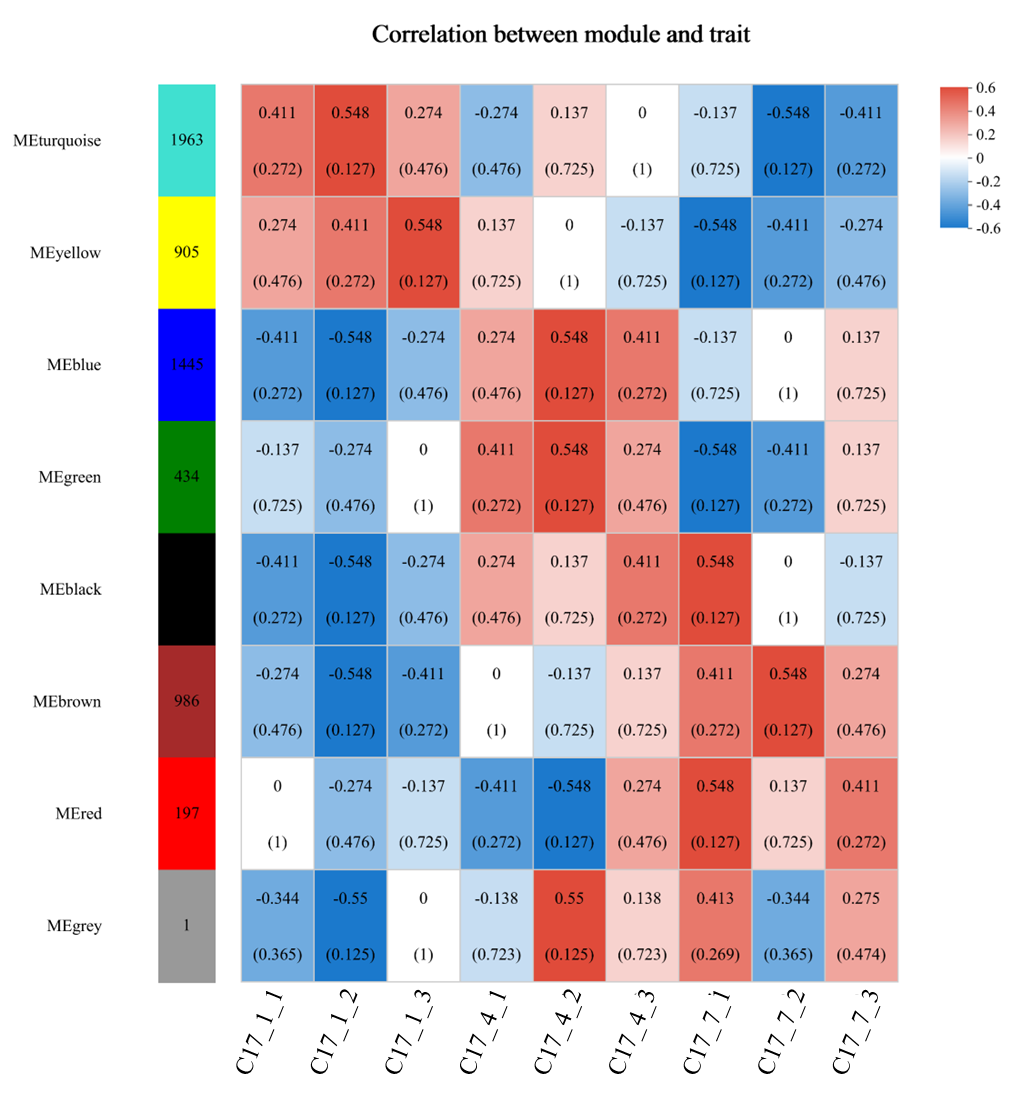
**

**FIG S6 Correlation between modules and traits. The horizontal coordinate represents different phenotypes/samples/groups, and the vertical coordinate represents different modules. The number of genes/transcripts in the module is indicated by a column of numbers on the left, and the correlation coefficient and significant P-value of the association between the module and the phenotype are indicated for each set of data on the right (in parentheses). A greater association is found with greater absolute values. A positive correlation is shown in red, and a negative correlation is shown in blue. See the numbers under the color bar at the bottom right for more correlation details. Usually, the most significant P-value and highest correlation module is chosen as the phenotypic characteristic module.**
